# Moving from In-Person to Digital Delivery Models of HIV Peer Support Services in Response to COVID-19: A Qualitative Study

**DOI:** 10.1007/s10461-025-04894-6

**Published:** 2025-10-10

**Authors:** Brent Clifton, Dean Murphy, Jeanne Ellard, Garrett Prestage, Nathanael Wells

**Affiliations:** 1 NAPWHA (National Association of People with HIV Australia), Newtown, Australia; 2 Australian Research Centre in Sex, Health, and Society, Melbourne, Australia; 3 Kirby Institute, UNSW, Kensington, Australia

**Keywords:** HIV peer support, HIV, COVID-19, Digital health, Peer support

## Abstract

COVID-19 saw a rapid shift in how community-based peer support programs were delivered. HIV peer support workers were required to work from home and community-based HIV and LGBTQ + organisations moved their support programs to digital platforms. Between May and September 2020, semi-structured interviews were conducted with individuals who worked (*n* = 17) or volunteered (*n* = 4) for community-based HIV and/or LGBTQ + health organisations. Interviews explored the impact of moving to digital service delivery on how peer support programs were delivered and the impact on peer support workers. We identified three overarching themes. Firstly, we highlight challenges with rapidly shifting to online service delivery, particularly as this shift limited opportunities for informal interactions between participants. Informal interactions were considered an important aspect of peer support programs. Secondly, the move to digital service delivery diminished opportunities for informal support between peer workers and their colleagues, just as they were also adjusting to a new and unfamiliar working environment. Thirdly, the removing of physical distance as a specific barrier to care opened new opportunities for engaging potential clients who may have previously had difficulties in accessing HIV support services. We argue that careful consideration is needed to address barriers specific to digital service delivery, including lack of access to appropriate technology and telecommunication infrastructure, as well as concerns about participants’ privacy.

## Introduction

COVID-19 saw a rapid and significant shift in health service delivery, with many services moving to delivering care through telehealth and/or digital platforms. Highly transmissible and with no cure or effective vaccine [1, 2], countries began implementing strategies to prevent the spread of COVID-19, including social distancing measures [3]. In response to COVID-19, Australian federal and state governments sought to minimise rates of transmission by banning foreign nationals from entering the country and implemented mandatory 14-day hotel quarantine for returning citizens [4]. Limitations on social gatherings were imposed, and those working in employment deemed “non-essential” were required to either stop working or where possible, work from home [5]. Deemed “non-essential,” community-based HIV and LGBTQ + health organisations were also required to shift their operations to a digital service delivery model almost overnight. In this paper, we explore the experiences of HIV peer workers in providing support to people living with HIV during the periods of COVID-19 working from home arrangements in 2020.

Peer support is recognised as an important aspect of HIV-related healthcare that improves the overall health and wellbeing of people living with HIV [6–8]. Although the nature of peer support varies across different health contexts, peer workers typically belong to the same communities they support and have a shared knowledge based on similar lived experience [7, 9–13]. Peer support programs have been shown to improve treatment uptake, adherence, and retention in care [9, 14, 15] and also decrease HIV risk behaviours [16]. Peer support programs can also build resilience against stigma among people living with HIV [17, 18] while also providing a sense of social connection and belonging to a wider HIV community [8, 19–21].

In response to working from home restrictions, many HIV and LGBTQ + health organisations providing care were required to rapidly incorporate videoconferencing into their day-to-day operations and service delivery [22–25]. The delivery of healthcare through digital platforms can have important benefits. The inclusion of digital service delivery can provide individuals increased autonomy over how they access healthcare services [26]. Digital service delivery can also remove physical location as a barrier for individuals to engage with health services [27]. However, challenges also exist when implementing digital health services. For example, some services may not be suitable to adapt to models of digital delivery [28]. Moreover, some service users may have concerns about potential breaches of privacy or a distrust of digital platforms, particularly those who belong to populations experiencing heightened forms of stigma and discrimination such as people living with HIV [28, 29].

While some research has explored the impact of COVID-19 on HIV testing, treatment and clinical care in Australia [30–34], the impact of COVID-19 on Australian community-based HIV and LGBTQ + support services remains less documented. In this paper we describe how the use of videoconferencing models of service delivery were integrated into service provision with a focus on the impact HIV peer support services. We also explore potential benefits and opportunities created by a shift to digital service delivery that may inform new initiatives for the delivery of HIV peer support in a post-lockdown context. In particular, we highlight important considerations for service delivery going forward, particularly as videoconferencing and telehealth become increasingly embedded in healthcare service provision.

## Methods

### Study Setting

This paper is based on data collected as part of a broader qualitative study that explored receiving and providing HIV diagnoses and HIV care among a cohort of people recently diagnosed with HIV in Australia. As part of the study, interviews were conducted with community-based HIV peer support service providers. These organisations provided various kinds of support to people living with HIV in Australia including group workshops, counselling services, educational seminars, individual peer navigation, social events, financial support, housing assistance, and alcohol and other drug support. The analysis for this paper is based on a subsample of participants who either worked for or volunteered as HIV peer support workers in community-based HIV organisations. The study design was approved by the University of New South Wales Human Research Ethics Committee.

### Eligibility and Recruitment

Eligible participants were those working in or volunteering for community-based HIV organisations, such as HIV/AIDS councils, and/or state and national organisations for people living with HIV. To be eligible, participants had to have been directly involved in providing peer support to people living with HIV at the time of their interview. Potential participants were recruited through formal and informal networks of people working in the Australian HIV sector. Potential participants were approached directly by members of the research team through publicly available contact details or through existing relationships and invited to participate in an interview. Participation was open to all those who were directly engaged in providing community-based HIV service delivery. A deliberate strategy of inviting participants from every Australian jurisdiction was employed. Prior to being interviewed, participants provided written consent. No compensation was offered for participating in this project as interviews were conducted as part of participants’ professional capacity.

### Data Collection

In-depth, semi-structured interviews were conducted by BC via the videoconferencing platform Zoom©. Interviews were chosen as the primary data collection method as they allowed for an in-depth and nuanced exploration of HIV peer support workers’ individual experiences in the initial stages of the Australian COVID-19 response. Interviews lasted between 30 and 60 min and were conducted between May and September 2020 during the early months of COVID-19 restrictions in Australia. An initial interview schedule was developed and then reviewed by members of the research team and an advisory group. Interviews explored the types of peer support offered, the impact of COVID-19 on how peer support programs were delivered, the forms of support that was offered to employees, and participants’ experiences of the shift to providing HIV peer support via digital platforms.

### Analysis

All authors were experienced qualitative researchers with extensive experience in researching the health and wellbeing needs of people living with HIV, and working with community-based LGBTQ + and HIV health organisations. Interviews were recorded and transcribed verbatim. Transcripts were de-identified, entered into NVivo software, and thematically analysed [35–37]. Following Braun and Clarke’s [38] approach to thematic analysis, our aim was capture in-depth and nuanced understandings of participants’ experiences. A codebook was developed by BC based on a close reading of a small sample of interviews and discussions with the research team. Subsequent interviews were then coded and the codebook revised as new codes were identified. Members of the research team met regularly to review and discuss findings as they arose. Analysis for this paper was conducted by BC. Pseudonyms have been used throughout to ensure participant anonymity.

## Findings

Interviews were conducted with 21 HIV community service providers across all Australian jurisdictions. Of these, 17 were paid staff who worked within community-based HIV organisations and the remaining four were volunteers. Five participants worked in New South Wales, four in Victoria, two in Queensland, two in Western Australia, two in South Australia, and one each in Tasmania, Northern Territory, and the Australian Capital Territory. The remaining three participants did not work for state-specific organisations and instead worked for peer-based organisations that provided support for all people living with HIV in Australia regardless of state. 18 participants identified as male and three as female. Most participants disclosed that they were living with HIV, although the question of participants’ HIV status was not asked as part of the demographic data collection.

The kinds of work participants did to support people living with HIV included one-on-one support, facilitation of group workshops, connecting clients to other non-HIV specific services, assisting clients navigate healthcare systems, provision of financial support, and psychosocial support services.

HIV community organisations provided a range of support services, including: one-on-one peer support and peer navigation, group workshops, education campaigns, social events, psychosocial support, alcohol and other drug services, financial support, and foodbank services. Until the emergence of COVID-19, the modes through which support had been delivered had largely remained static, with service delivery primarily centred on an in-person model. In the analysis that follows, we describe three overarching themes: challenges in adapting service provision to online service provision; ensuring the mental wellbeing of peers; and new opportunities for future service delivery.

### Challenges in Adapting to Online Service Provision

The emergence of COVID-19 saw rapid and major changes in how services were offered by many community-based HIV organisations. An almost overnight migration from office-based working environments to working from home saw a proliferation in the use of video conferencing software [39]. This rapid shift to working from home presented challenges in adapting some support programs to a digital delivery model, particularly in-person support groups and workshops. Nate explained:

Knowing how long it was going to be before we could run an onsite program and not my [newly diagnosed] workshop, I thought to myself: “what could we do in the meantime?: a medical session where it’s basically all about HIV monitoring and treatments. It’s a PowerPoint presentation delivered by [a colleague]. It’s not really about peer support. It’s not really about sharing or anything like that. It’s just basically information based.” And so I thought, “that could easily be a webinar.” And we did that.

Some events such as webinars and educational sessions could be easily adapted to an online format with relative ease. The provision of more interactive group programs, however, proved more challenging to adapt. While not discounting the value of webinars and educational sessions, Nate believed that these could not elicit the same sense of connection or support as in-person peer group programs.

Creating a sense of belonging and connection was a highly valued aspect of in-person peer support programs and workshops for participants. While video conferencing went some way to maintain a sense of connection, participants commonly felt this form of connection to be limited. Stevie, for example, stated:

Our retreat workshops for newly diagnosed people, social events, seminars, all of those sorts of things we’ve had to either move online, which we have done some of them really successfully. But there are certain things you just can’t do in a webinar or in an online environment. So, they just had to be put on hold.

Reflecting on digital service delivery, Brett commented: “*It’s all less personal in a sense.*” Similarly, Kenny reflected on the shift of workshop to digital models, stating: “*Someone newly diagnosed would usually have access to in-person support*,* seeing someone in-person*,* or attending a workshop. Right now*,* they’re missing that.*” While some services such as webinars had been successfully adapted for the online environment, participants commonly felt that opportunities for interpersonal connection were more limited in a digital environment. In particular, opportunities for more informal interactions, such as during coffee breaks and/or socialising could not be reproduced in an online environment.

Throughout the COVID-19 pandemic, many of the services offered by community-based HIV organisations evolved to suit delivery through video conferencing platforms. While this enabled the work of service providers to continue, it also presented challenges. Kyle stated:

There are some challenges with [video conferencing]. Like picking up on the verbal cues … You can’t see people’s actual presentation and for me when I’m talking to somebody – particularly who’s recently diagnosed – I’m looking for a lot of non-verbal cues in terms of what’s going on for them. That’s not so bad one-on-one but group work? Oh man, that’s full on.

Gauging visual cues such as body language was a way for peer facilitators to assess how clients might be coping and for Kyle, was considered relatively straightforward in face-to-face settings. As many of these cues were lost through video conferencing, participants became more dependent on both verbal and facial cues. The loss of body language as a measure of how clients were coping added extra complexity to the work of providing clients with appropriate support.

Confidentiality has long been the foundation of services offered by community-based HIV organisations who strive to create an environment in which people living with HIV can access services while maintaining their privacy and mitigating against the risk of unwanted HIV disclosure. Maintaining confidentiality in digital spaces was regularly described as a substantial challenge, as Nate commented:

People are afraid [of] their identity being disclosed online because there’s no control, there’s less control online. Whereas physically you walk into a building and into a room, you have more control over that and you can see who’s in the room. Whereas online, some people think to themselves – and I’m thinking from the perspective of a newly diagnosed person who’s really cautious about their privacy – going online you don’t know. There’s less control. You don’t know who’s watching, who’s listening. It’s just slightly different. Particularly for a newly diagnosed person who’s extra cautious.

Workshops and support groups, particularly those for newly diagnosed PLHIV, are contexts in which confidentiality is crucial as it is in these settings that individuals interact with other members of the community. While participants in group workshops can never be completely protected against the risk of unwanted disclosure, they are afforded a greater sense as to who else is participating by virtue of being in the same room. Some participants perceived digital environments as contexts in which there was less control, particularly given the possibility of non-participants being able to observe or overhear program participants. Nate did not explicitly state that he had actually experienced an individual decline to participate in a digital group workshop owing to concerns about confidentiality. Nonetheless, his perception of heightened confidentiality concerns highlights the additional factors that peer workers were required to consider in the shift to digital program models.

### Ensuring the Mental Wellbeing of Peers

The nature of peer support work means that inevitably, participants regularly provide support to people living with HIV who were going through complex and distressing experiences, experiences that became exacerbated by COVID-19 restrictions. At the same time, however, participants themselves were also experiencing rapid changes in their own lives. Reflecting on the speed with which these changes occurred, Eric stated:

It all happened really quickly. In terms of the organisation, everyone was literally told to go home, and we had to sort of start building our lives – our work lives – again.

Eric highlighted the disruption caused by COVID-19 such that he experienced the adjustment to working from home arrangements as a need to “rebuild” his work life again. Similarly, some participants described the disruption to existing routines, both work and personal, as a particular challenge brought about by COVID-19:

I felt disruption to some of my healthier habits. I guess I’ve given myself permission to eat trashier food, maybe turn Tuesday night into a home party when I was pretty good [at not partying] Sunday through Thursday previously (Brett).

Participants commonly described the shift to working from home as impacting their own mental health and wellbeing, adding an additional challenge to providing support to clients of their services. Just as participants were experiencing their own challenges in adjusting to new uncertainties brought about by COVID-19, they were also supporting clients who were often experiencing complex and challenging situations related to their living with HIV and the impact of COVID-19 on day-to-day life.

For some participants, working from home arrangements also saw a blurring of boundaries between work and home life. Stevie commented:

All of the peer navigators are working from home which is difficult for them because it means that their home, which is usually their sanctuary, becomes their workplace where they are dealing with really difficult situations. So, getting that break between home and work where they can, you know, feel like at least on a spiritual level or something, leave their work behind in the workplace and come home to their sanctuary.

Stevie described the home as a “*sanctuary*,” a place in which peer support workers could recover from what was often characterised as difficult and challenging work. For Stevie, the shift to working from home arrangements was described as a spiritual challenge for HIV peer support workers. Without a break between leaving work and going home, peer navigators were almost always *at* their workplace, even if not actively working, creating an additional challenge of separating work and home.

Peer support workers experienced rapid changes to their working environments throughout the COVID-19 pandemic. This shift limited opportunities for regular, informal social connections between HIV organisation employees. Reflecting on the initial separation from his colleagues, Ben stated:

[I] think the first couple of months it definitely rattled the staff, I think is a fair way to put it. And it probably still is in many ways … because we are not just colleagues. A lot of us are really close friends as well.

The social bonds Ben felt with work colleagues went beyond simply those of work relationships and instead, Ben considered many of his colleagues to be close, personal friends. Given these close bonds, the workplace was also an important source of social connection, a source of connection that became more challenging to maintain with COVID-19 restrictions. The challenges of maintaining close connections during COVID-19 restrictions meant that participants were making their own adjustments and dealing with their own anxieties about the pandemic without their usual face-to-face workplace interactions.

Ben’s experience was echoed by Brett who described losing: “*The ability to giggle with each other and say*,* ‘Hey! What’s up? What are you doing today?’ All that is now absent when you’re at home.*” To mitigate against feelings of social isolation, Brett went on to describe his organisation as:

Leveraging things like Microsoft Teams … we have a space where it’s more official about work. But we also have a space to share memes, and videos, and songs, or whatever.

Brett described a delineation between more structured, official workspaces and the creation of dedicated, less formal spaces in which employees could maintain social connection with their colleagues.

### New Opportunities for Future Service Delivery

Just as COVID-19 restrictions caused significant challenges for community-based HIV organisations, it also opened new possibilities for how organisations engaged with communities they had previously not reached. For some participants, there was a sense that the shift to digital service delivery was a welcome change, a change that they hoped would continue even as lockdown measures eased. Nate commented: “*We certainly want to continue our online engagement*,* which is something we should be doing anyway.*” Similarly, Joseph welcomed some of the changes instigated by COVID-19:

COVID-19 broke down a barrier between some of this type of online interaction … it normalised it and so that was some of the bi-products, some of the things that came out of COVID.

Here, Joseph described COVID-19 as almost forcing HIV organisations to adapt and find new and innovative ways of providing support to people living with HIV. Rather than being characterised as a shift specific to COVID-19, however, both Joseph and Nate suggested that this was a shift that organisations should have already been making and one they hoped would continue into the future.

The shift to digital service delivery removed location as a specific barrier to accessing peer support, creating new possibilities for those unable to attend in-person programs to engage with community-based HIV organisations. Stevie commented:

We have had people who had never attended before who are attending now. And that may be because they were rural, or they have a disability, or they just have a busy life and they can’t normally attend.

While delivering services through video conferencing platforms presented some challenges for service providers, it also removed some barriers to accessing these services, particularly those for whom physically attending peer support was more difficult.

Cognisant of the potential negative impact COVID-19 might have on people’s psychosocial wellbeing, some participants described actively engaging with other allied health services to strengthen referral pathways. Stevie recalled:

We also let our referrers know that we were … actually anticipating this [negative impact] and encouraged them to make referrals for people they were concerned about, who are socially isolated, or who are likely to be particularly vulnerable because of COVID-19 and the restrictions that are being placed on them.

Prior to COVID-19, one focus of peer support programs was on assisting people living with HIV navigate sometimes complex healthcare systems. In some cases, peers would even accompany newly diagnosed people living with HIV when visiting healthcare providers. As described by Stevie, some community-based organisations leveraged existing networks to encourage healthcare providers to refer clients who might be experiencing increased vulnerabilities due to COVID-19.

## Discussion

To our knowledge, this is the first study to explore in-depth the experiences of Australian HIV peer support workers providing support to people living with HIV during the COVID-19 pandemic. The incorporation of digital service delivery was seen across the healthcare sector, with telehealth models of care crucial in ensuring the continued delivery of health services throughout the COVID-19 pandemic [23, 40, 41]. The incorporation of digital modes of service delivery presented challenges, particularly as service organisations had limited time to adapt their programs. Despite these challenges, the digital platforms enabled HIV organisations to maintain a degree of continuity in the provision of peer support services.

Concepts of community development [42] and empowerment [43, 44] have been, and continue to be, central to the provision of support and programs offered by these HIV organisations. The concept of community development is underpinned by an ethos of working collaboratively with individuals affiliated by a common condition, social justice, and capacity building [42]. The programs offered by community-based HIV organisations have evolved in line with shifts in the HIV epidemic. For example, the development of effective HIV treatments have seen a shift of HIV as a fatal illness to being a chronic, manageable condition [45]. In turn, the focus of community-based HIV support programs has shifted to supporting individuals to live long-term with HIV. Additionally, while peer-based HIV and LGBTQ + programs continue to be influenced by their history of grassroots advocacy for marginalised populations, HIV and LGBTQ + health organisations have become more professionalised over time. The move to delivering peer-based HIV support programs through digital platforms represents another shift in HIV service delivery.

Our findings highlight important considerations when conceptualising and designing HIV support services to be delivered through digital and telehealth models of care. As noted by some participants in our study, working from home arrangements necessitated the provision of support to people with often complex needs to be delivered in peer support workers’ own homes. For some participants, the home space had previously been imagined as a space distinct from their work and the challenges in helping others with sometimes complex psychosocial needs. Moreover, working from home also removed opportunities for informal interactions with other colleagues [46], interactions that were valued as an important form of support.

Strong feelings of belonging to a community of peers has been associated with lower levels of burnout and higher levels of social support [47]. Similarly, Anderson and colleagues [48] found that a strong sense of social support provides opportunities for peers to debrief. These findings were echoed by participants in this study, who indicated that prior to the shift to digital service delivery, interactions with colleagues within the physical workplace provided opportunities for informal support. In shifting to the digital service delivery (whether fully or partially), it is necessary that organisations consider strategies to maintain opportunities for informal interactions among peer workforces. Similarly, there is a need for organisations to ensure peers have access to opportunities for ongoing training and formal support. However, such training and support should extend beyond simply service provision and debriefing, and also target peer mental health more broadly.

Aligning with previous research [24], participants in this study reported that by removing physical location as a barrier to service access, the shift to digital service delivery created opportunities for service providers to engage with people they had previously not engaged with. Importantly, however, this shift did not necessarily eradicate all barriers to service access. Indeed, digital service delivery necessitated consideration of the potential for new barriers to access HIV support services. While delivering services online can be convenient for many, it is necessary to also be cognisant of a potential “digital divide”: accessing digital support services requires individuals to also have access to the appropriate technology and telecommunication infrastructure [24, 49–51] while also being confident and able to use different forms of technology [52]. A lack of access to telecommunication technologies often disproportionately affects those who are more socially and economically marginalised [53]. It is essential that in-person delivery is also maintained and not entirely replaced by digital service delivery [28].

While promising, shifting services to digital platforms also requires careful security considerations. Echoing previous research [24, 51], we found that some participants raised the potential for some clients to be concerned about privacy and who other participants in group sessions were. This concern was amplified owing to the sensitive nature of topics discussed and the potential for unwanted disclosure of their HIV status. Houser and colleagues [54] suggest organisations that deliver health services through digital platforms require the use of a password by their clients. While not a panacea, this strategy may go some way to mitigate against some privacy concerns.

When designing online HIV peer support programs, there is a need to consider what types of programs are best suited for digital service delivery models. Our findings align with previous research that identifies some programs may not easily be adapted to, or appropriate for, the delivery of healthcare through digital platforms [28]. While participants identified informational, webinar-type sessions as easy to adapt for online delivery, adapting programs such as group workshops was less appropriate. One of the benefits of peer-based HIV support workshops is in building, both formally and informally, a sense of belonging and community among people living with HIV [8]. Communication through videoconferencing platforms limits opportunities for less formal interactions between workshop facilitators and their clients, while also limiting informal interactions between workshop participants themselves. In turn, opportunities to build social connections between other people living with HIV may be limited by shifting programs to digital delivery models.

Moving forward, mixed models of service delivery that include both digital and in-person mediums have the potential to become embedded as an important and standard aspect of healthcare delivery [55]. Ongoing evaluation of digital models of care will also be important in understanding the kinds of support that best meets the needs of community members. Ongoing evaluation is particularly important given demographic shifts in new Australian HIV notifications, whereby a significant decline in notifications among Australian-born GBM has been observed while rates among migrant GBM and heterosexuals remains stable [56]. Moreover, it is crucial that peer support workers are provided with the appropriate skills, training, and resources to support people living with HIV through new modalities of service delivery.

### Limitations

Our findings reflect a specific moment of the COVID-19 pandemic. Interviews were conducted in the first weeks of the pandemic, a period marked by rapidly (sometimes daily) changing restrictions. During this period, organisations were having to respond to changing restrictions, often with little or no warning or time to prepare. Interviews were also conducted prior to extended restriction periods in Australia’s two most populous states, Victoria and New South Wales. Had interviews been conducted during these periods, they would likely have uncovered different concerns. Moreover, interviews conducted during this period would have also enabled an exploration of how community-based organisations had embedded digital service delivery as part of their routine standard of care.

As this study was conducted during a period in which organisational responses to COVID-19 were rapidly changing, interviews did not explicitly explore the funding models and forms of training offered to peer workers in response to COVID-19. We are therefore unable to comment on the effectiveness of different funding and training models responding to COVID-19 by different HIV peer support organisations.

Interviews were only conducted with individuals who were actively involved in providing peer support to people living with HIV and not conducted with those in management positions. As such, we are unable to provide a more holistic perspective of the *how* and *why* certain decisions were made in the shift to digital service delivery. Similarly, the majority of participants held paid positions within their organisations with only four participants being volunteers, potentially influencing the perspectives represented.

## Conclusion

COVID-19 and the associated restrictions on movement caused significant disruption to traditional models of HIV healthcare delivery. Healthcare service providers, including community-based HIV organisations, rapidly incorporated telehealth and digital service delivery into their standard service provision models. Removing physical distance as a specific barrier to care opens new opportunities for engaging potential clients who may have previously had difficulties in accessing HIV support services. However, careful consideration is also needed to address barriers specific to digital service delivery, including lack of access to appropriate technology and telecommunication infrastructure, as well as concerns about participants’ privacy. While adopting telehealth models of HIV support offer exciting opportunities for increasing engagement with community-based support services, there is a continuing need for in-person programs, particularly as in-person support programs provide opportunities for people living with HIV to connect less formally with other people living with HIV. In the context of limited funding for HIV support services, it is necessary to ensure community-based HIV organisations are appropriately resourced to meet the needs of all people living with HIV.

## Data Availability

Participants did not provide consent for their data to be made publicly available. Due to the sensitive nature of this research, supporting data is not available.
